# Hybrid YSGOA and neural networks based software failure prediction in cloud systems

**DOI:** 10.1038/s41598-024-67107-5

**Published:** 2024-07-11

**Authors:** Ramandeep Kaur, Revathi Vaithiyanathan

**Affiliations:** 1 Assistant Professor, Department of Computer Science & Technology; Research Scholar, Department of Computer Science & Engineering, Dayananda Sagar University, Bangalore, India; 2 Associate Professor, Department of Computer Science and Engineering, Dayananda Sagar University, Bangalore, India

**Keywords:** Software failure detection, Deep learning, Feature selection, Optimization, Cloud computing, Engineering, Mathematics and computing

## Abstract

In the realm of cloud computing, ensuring the dependability and robustness of software systems is paramount. The intricate and evolving nature of cloud infrastructures, however, presents substantial obstacles in the pre-emptive identification and rectification of software anomalies. This study introduces an innovative methodology that amalgamates hybrid optimization algorithms with Neural Networks (NN) to refine the prediction of software malfunctions. The core objective is to augment the purity metric of our method across diverse operational conditions. This is accomplished through the utilization of two distinct optimization algorithms: the Yellow Saddle Goat Fish Algorithm (YSGA), which is instrumental in the discernment of pivotal features linked to software failures, and the Grasshopper Optimization Algorithm (GOA), which further polishes the feature compilation. These features are then processed by Neural Networks (NN), capitalizing on their proficiency in deciphering intricate data patterns and interconnections. The NNs are integral to the classification of instances predicated on the ascertained features. Our evaluation, conducted using the Failure-Dataset-OpenStack database and MATLAB Software, demonstrates that the hybrid optimization strategy employed for feature selection significantly curtails complexity and expedites processing

## Introduction

Cloud computing has emerged as a dominant paradigm in the modern era, providing scalable and flexible computing resources to meet the demands of various applications and services^1^. As cloud computing continues to gain popularity, ensuring the reliability and stability of software systems deployed in cloud environments becomes crucial. Cloud software systems, despite their advantages, are not immune to failure. Due to the complex and distributed nature of cloud environments, failures can occur in unanticipated ways, often leading to unexpected consequences^2,3^. The interconnectedness of cloud software systems means that a failure in one component can have a domino effect, impacting other dependent components. This cascading propagation of errors can be challenging to predict and mitigate, as failures may manifest in unforeseen ways and spread rapidly across the system. A failure in a single component, such as a server or a network element, can trigger a chain reaction that affects multiple other components, potentially leading to system-wide disruption^4^.

Before deploying a software system in a cloud environment, cloud system designers must have a clear understanding of how the system will respond and behave in the face of failures. This understanding is essential for proactively preventing service disruptions and ensuring the reliability of the overall system. Fault injection in cloud computing involves intentionally introducing faults or failures into a cloud-based software system under controlled conditions^5^. This can include simulating resource exhaustion, inducing software defects, causing network connection loss, or other types of faults. The purpose of fault injection is to observe and analyse how the cloud system reacts to these simulated failures and to gather data on the system's behaviour and performance in such scenarios. By injecting faults, researchers and engineers can assess the system's resilience, evaluate its failure recovery mechanisms, and identify potential vulnerabilities^6^. During fault injection experiments, a significant amount of data is generated, including hundreds of thousands of events and execution traces related to failure occurrences. This data provides valuable insights into the behaviour and characteristics of software failures within the system. However, the challenge lies in extracting meaningful information from this vast amount of data to identify recurrent failure modes and their relative frequencies.

Failure mode analysis analyses the collected data from fault injection experiments or real-world failure incidents in cloud computing^7^. It involves systematically examining the failure events and traces to identify recurring failure modes or patterns. Failure mode analysis aims to pinpoint the specific types of failures that occur frequently and have a significant impact on the cloud system's performance and availability. By analysing the failure modes, cloud system designers and operators can gain insights into the root causes of failures, identify common failure patterns, and assess the relative frequency and severity of different failure modes^8^. This analysis helps guide the development of effective failure management techniques and strategies. The performance of current software failure analysis models is determined by purity metric that typically signifies the proportion of software failures that can be attributed to specific, identifiable causes. It is a measure of how well the model can isolate and categorize failures based on their root causes, rather than treating them as random or unexplainable occurrences. The high purity indicates that the model is effective in diagnosing and categorizing failures accurately. On the other hand, low purity suggests that many failures are not being accurately attributed to specific causes, implying potential gaps in the model. However, Failure mode analysis is a complex and time-consuming task due to the abundance and intricacy of failure data in cloud systems. The non-deterministic behaviour of these systems further complicates the analysis process, as it introduces noise into the failure data through random variations in the timing and sequencing of system events. Therefore, while developing a software failure diagnosis model, the developers should focus on improving purity value to ensure its effective working.

Consequently, failure mode analysis algorithms must be robust enough to handle such data imperfections and account for the noise. To address these challenges, unsupervised machine learning (ML) approaches, such as clustering and anomaly detection, were commonly employed in failure mode analysis. These techniques aim to uncover patterns and anomalies within the failure data. However, there are still limitations associated with these approaches in terms of their effectiveness in handling the unique characteristics of cloud failure data. To improve the suitability of failure data for analysis in ML models, feature engineering is often employed. Feature engineering involves selecting and transforming relevant features from the failure data to enhance the performance of analysis algorithms. This step helps in capturing meaningful information and reducing the impact of noise on the analysis results. In recent years, the integration of deep learning techniques has shown promising potential in the field of failure mode analysis. Deep learning algorithms, such as deep neural networks, can automatically learn and extract complex patterns and representations from the failure data, alleviating the need for manual feature engineering. These techniques have demonstrated improved accuracy and effectiveness in detecting and analysing failure modes in cloud systems^9^. Deep learning models have found their application in all major and minor domains of the current world, especially in the medical domain. The authors in^10^ discussed the role of explainable AI-based techniques in understanding their working mechanism for predictions. By doing so, they were able to form a trust for system predictions, resulting in accurate disease detection. They categorized different XAI-based techniques along with their current challenges and future directions. Also, the DL and fuzzy systems approach was proposed in^11^ for detecting arrhythmia through ECG signals. They utilized CNN for extracting features and a fuzzy clustering approach was used for classifying heart diseases found in a particular ECG signal. Moreover, it was also analysed that the efficacy of AI models can be enhanced significantly with the integration of evolutionary algorithms. Since their inception, optimization algorithms have been used for solving various multi-objective problems, and biomedical applications. For instance, the authors in^12^ proposed an approach in which they aimed to enhance Surrogate-assisted evolutionary algorithms (SAEAs) for solving complex and intricate optimization problems. Similarly, the authors in^13^ worked on solving multi-objective optimization problems falling under the MINLP category by using a compressed coding scheme. Additionally, a ResNet50 and CNN-based acute lymphoblastic leukemia (ALL) detection model was presented in^14^, whose performance was improved with the help of a hybrid PSO optimization algorithm.

In this research paper, we propose an effective and efficient software failure detection model for cloud environments. The proposed model utilized DL-based Neural Networks (NN) in the classification phase to overcome the shortcomings of traditional ML approaches. Furthermore, a hybrid feature selection technique based on YSGA and GOA is also proposed for selecting only optimal and discriminative features from the given dataset. The efficacy of the proposed approach is analysed and validated on a publicly accessible Failure-Dataset-OpenStack dataset, available on GitHub. Moreover, we evaluated and compared the performance of the proposed approach with standard approaches in MATLAB software in terms of their purity value. A detailed description of the results is given in Section “Yellow saddle goat fish algorithm (YSGA)” of this paper.

The remaining section of this paper is: Section “Literature review” discusses recently proposed literature, followed by the problem statement. Section “Proposed model” gives an overview of the proposed model and its working methodology. Section “Yellow saddle goat fish algorithm (YSGA)” discusses the experimental setup and performance analysis of the proposed model and finally, a conclusion is written in Section “Grasshopper optimization algorithm (GOA)”.

## Literature review

In the realm of software failure prediction, numerous models and techniques have been presented in the literature. In this study, we leverage a diverse set of keywords like Software failure prediction, Software Quality, Neural Network, etc. By employing a comprehensive approach, we aim to provide a comprehensive overview and analysis of the existing research landscape on software failure prediction. In^15^, they utilized a DL-based Deep Embedded Clustering (DEC) approach wherein, an autoencoder was used to reduce the dimensionality and inter-cluster variance. The performance of the suggested approach was validated on original failure data and in integration with the anomaly detection pre-processing method. Results revealed that their suggested approach achieves good and comparable results in terms of purity values. Moreover, the authors in^16^, proposed a GA and DNN-based software failure prediction model. The approach utilizes Improved GA for Feature selection and DNN for classification purposes on the PROMISE database. Results revealed that the highest accuracy of 98% was achieved for the PC4 database, followed by 97.96%, 97.82%, and 97.59% on the PC3, KC1, and CM1 databases. In^17^, researchers explored 4 predefined AI algorithms (ANN, RNN, GRU, and LSTM) for predicting software failures by employing a time series software failure database. They trained every algorithm on the given database to anticipate software failure time after a specific number of corrective modifications. Results showcased that suggested LSTM model was outperforming the other three approaches. Similarly, the authors in^18^, presented a unique technique in which fault injection and anomaly detection were clubbed together for recognizing failure attributes. They analysed the model on the OpenStack CC platform which showed significant improvement in the accuracy of failure analysis, with less computational cost.

Eight distinct embedded approaches were used in^19^, to compute an n-dimensional numeric vector for fault reporting. To uncover pertinent characteristics and address the disparate sample distribution across classes, they employed feature selection approaches and data sampling techniques. The experimental findings from six studies showed that using current techniques improves the accuracy of fault degree of severity prediction models. Moreover, to make it easier to find and understand failure modes, the authors in^20^ developed an innovative approach (fault injection analytics) that uses unsupervised ML on execution records of the injecting system. The model was analyzed on the OpenStack CC platform, upon which effective results were achieved with loess computational cost. Similarly, the authors in^21^, reviewed the impact of DL in software defect prediction. They utilized four entirely different configurations of MLP neural Networks on a software failure repository. Results revealed that the MLP model with 2 hidden layers of 25 and 5 neurons respectively shows effective results for software failure detection. The researchers in^22^, provided a reliable k-means clustering and feature selection into a single framework for choosing precise features and enhancing the efficiency of clustering. Moreover, they also integrated the l2.p-norm into the objective function to increase the robustness of the system. Findings from experiments show that when compared to other approaches, the proposed strategy performs more reliably and effectively on reference datasets. In^23^, experts reviewed some supervised ML methods that were employed in software failure predictions and assessed their effectiveness in multi-standard databases. They investigated the parameter values of various classifiers and the advantages of using dimensionality reduction techniques like PCA and ensemble learning. Findings from the results revealed that PCA doesn’t have much impact on prediction systems, although parameter adjustment significantly improved the accuracy of classes, particularly with ANN. The most successful outcomes come from applying ensemble learning techniques like bagging getting 95.1% accuracy on Mozilla dataset, and voting 93.79% accuracy on kc1 dataset.

In addition to this, the authors in^24^ introduced a Cuckoo search-based software failure prediction model that was trained and tested on a software metrics database. Their basic objective was to analyze how well the CS algorithm performs while extracting important features from the given dataset. Through simulating results, it was proved that CSO not only improved accuracy but also reduced the intricacy of the model. Again, the authors in^25^ utilized hybrid GA and PSO for computing the threshold values from ten open-source databases and four procedural software datasets. Furthermore, researchers in^26^ proposed a GA-PSO and bagging technique-based software failure prediction model. They implemented the GA-PSO algorithm for selecting features from datasets and the class imbalance problem was mitigated using the bagging technique. Results showcased that by using the proposed technique model improved the accuracy of the failure diagnosis. Yet again in^27^ a hybrid metaheuristic model including GSO and GA was proposed with kernel-based SVM for predicting software failures. Results revealed that their model was able to detect faults with high accuracy.

Despite significant advancements in software failure prediction systems for cloud systems, several challenges and limitations persist. One of the primary issues is the inherent complexity and variability of cloud environments, which often introduce noise and uncertainty into the data used for prediction. Additionally, the dynamic nature of cloud systems poses difficulties in capturing and modeling the evolving behaviors and interactions of various components. Another challenge is the limited effectiveness of traditional feature selection techniques in identifying relevant and informative features for prediction. To address this, the integration of optimization algorithms has been explored to enhance feature selection and improve the performance of prediction models. However, the majority of this optimization algorithm suffers from slow convergence rates and a tendency to get trapped in local minima, which further increases the complexity and computational time of the model. Keeping these findings in mind, a new and effective failure prediction model must be proposed that can overcome such limitations.

## Proposed model

This portion of the paper gives a detailed overview of the proposed software failure detection model in a cloud environment. In the proposed work, we present a novel approach that combines hybrid optimization algorithms for feature selection with Neural Networks (NN) for classification. The key objective of our work is to improve the purity value of the proposed approach for different workloads. To combat this task, a failure dataset is taken from GitHub which comprises three workloads i.e., STO, NET, and DEPL, and different input and target variables. Before implementing any technique on the given dataset, it is important to separate the input and target variables to enable the model to generalize patterns and relationships between the two during the training phase. After this, feature selection and classification techniques are implemented to predict the failure. However, we have seen from the literature that researchers were using single optimization techniques for selecting features in the network, but a majority of these optimization algorithms either have slow convergence rates or get trapped in local minima, degrading the overall performance of the network. Therefore, to add the concept of novelty in the proposed work, we have used two effective optimization algorithms, namely; Yellow Saddle Goat Fish (YSGA)^28^ and Grasshopper Optimization algorithm (GOA)^29^ together to enhance the feature selection process. By integrating YSGA and GOA, we utilized the strengths of both algorithms in exploration and exploitation, leading to faster convergence, higher-quality solutions, and greater adaptability to complex problems. Moreover, an optimal or near-optimal solution was found by exploring the search space more effectively and efficiently thus overcoming problems faced in traditional single-algorithm approaches. By combining different algorithms, hybrid optimization can exploit the complementary characteristics of each algorithm, leading to better feature selection outcomes.

## Yellow saddle goat fish algorithm (YSGA)

This algorithm is one of the recently proposed optimization algorithms that was Zaldivar in^28^. The algorithm is a computational method inspired by the foraging behavior and movement patterns of the yellow saddle goatfish (Parupeneus cyclostomus). This fish species is known for its unique way of searching for food in sandy ocean bottoms, using its barbels to detect prey buried in the substrate. The algorithm mimics these behaviors to optimize complex problem-solving tasks, particularly in domains requiring efficient search and exploration techniques. It employs a combination of local and global search strategies, where the local search mimics the goatfish's precise, localized probing for food, while the global search replicates its broader, more exploratory swimming patterns. The algorithm iteratively adjusts the balance between local and global search efforts to dynamically adapt to the problem landscape, ensuring thorough exploration while honing in on optimal solutions. Its applications span various fields, including engineering design, logistics, and artificial intelligence, where it excels in handling multimodal functions and large, complex datasets. By leveraging the biological principles of the yellow saddle goatfish, this algorithm provides a robust and versatile tool for tackling optimization challenges with high efficiency and accuracy.

## Grasshopper optimization algorithm (GOA)

GOA is another famous optimization algorithm that was proposed by Mirjalili S in^29^, depicting the swarming behaviour of grasshoppers. Grasshoppers exhibit complex, collective movement patterns, particularly during their nymph stage when they form large swarms that move in a coordinated fashion. These movements are primarily driven by a combination of attraction, repulsion, and a tendency to move towards a target direction. In the context of optimization, a population of grasshoppers represents potential solutions, with their positions being updated iteratively based on social interactions, target attraction, and environmental factors. The mathematical framework of GOA includes components that simulate these dynamics, ensuring a balance between the exploration of new areas and the exploitation of known good solutions. This balance shifts over iterations, with early stages emphasizing exploration and later stages focusing on exploitation to refine solutions. The GOA is versatile and efficient, capable of handling large, complex, and multimodal search spaces, making it applicable to various fields such as engineering design, machine learning, scheduling, robotics, and network design. Its robustness and flexibility allow it to adapt to different optimization problems and constraints, providing a powerful tool for finding global optima while avoiding local minima.

In the next phase of work, we have employed the Neural Network (NN) model for identifying failures in the cloud computing environment. Neural networks are commonly used for classification in software failure prediction models due to their ability to learn complex patterns and relationships in data. Software failure prediction involves determining whether a software system or component is likely to fail or experience abnormal behaviour. This task requires analysing various input features and making accurate predictions based on those features. NN can automatically learn relevant features from the input data during the training process. Instead of relying on manual feature engineering, which can be time-consuming and subjective, neural networks extract meaningful representations from high-dimensional data. This ability is particularly beneficial when dealing with diverse and evolving software failure patterns. Furthermore, NN has built-in mechanisms, such as regularization techniques and robust activation functions, that help them handle noisy input and maintain stable performance even in the presence of uncertainties.

### Hybrid YSGA_GOA feature selection technique

**Figure 1:**
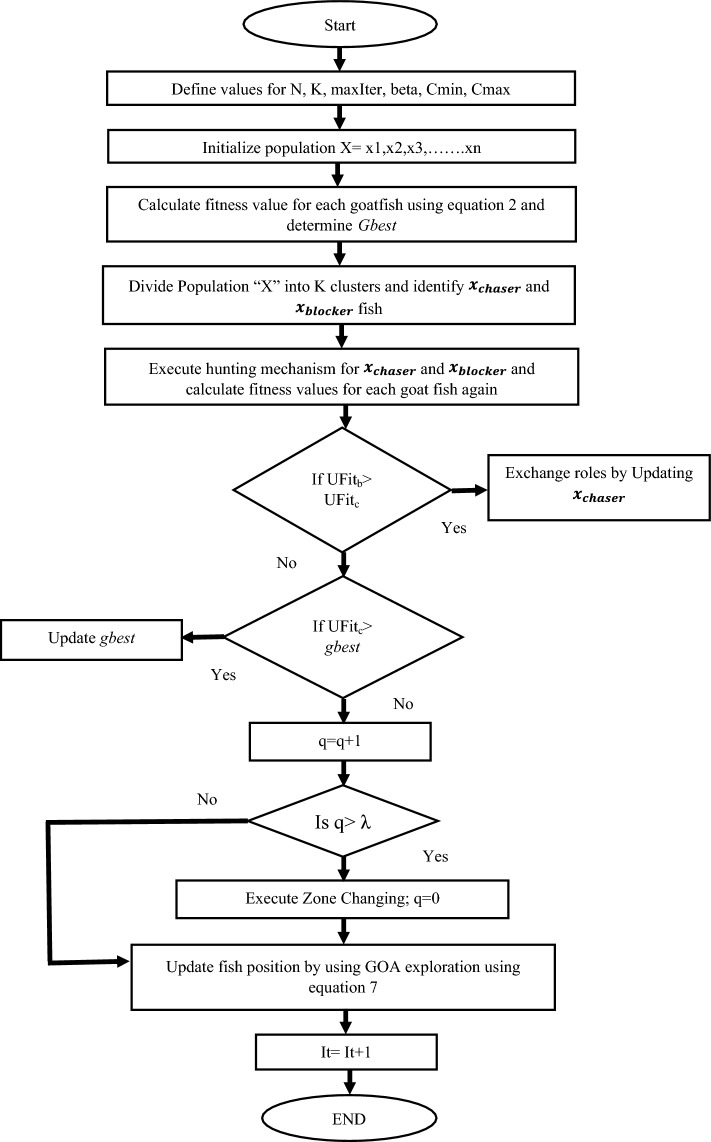
Flowchart of HYSGA-GOA.

In the proposed work, two nature-inspired optimization algorithms i.e., YSGA and GOA are hybridized for selecting features in a software failure prediction model that combines their respective convergence rates, resulting in an enhanced and efficient Feature Selection process. The convergence rate abilities of YSGA and GOA in feature selection play a crucial role in optimizing the software failure prediction model. One of the important reasons for using YSGA and GOA in the proposed work is to maintain a balance between exploration and exploitation phases. The convergence capabilities of YSGA allow for fast and effective exploration of the feature space, while GOA's convergence strengths enable efficient exploitation and fine-tuning of the selected features. These combined advantages of both exploration and exploitation phases give enough opportunity to the given algorithm to solve the NP-hard problem specifically the selection of features from the dataset. It also reduces the issue of getting stuck in local optima for the algorithm while exploring the solution for the given problem. By converging towards optimal solutions at an accelerated rate, the hybrid approach reduces the time and computational resources required for feature selection, allowing for faster deployment of reliable failure prediction models in cloud computing environments. The flowchart of HYSGA-GOA feature selection mechanism is shown in Fig. 1.

The YSGA algorithm employs the pack hunting behaviour of animals to capture the prey. They start hunting in two groups chaser fish and blocker fish. It must be noted here that YSGA considers the total number of fishes as population size and hunting area as search space respectively. On the other hand, GOA mimics the foraging behaviour of grasshoppers for seeking food. It has been demonstrated that this technique is effective in resolving globally constrained and unconstrained optimization issues and hence is used in the proposed work as well. The two algorithms are combined to select features from the given dataset in the proposed model so that the efficiency and effectiveness of the fault prediction model are enhanced. It undergoes many phases, as shown in Algorithm 1.

#### HYSGA_GOA parameters

At the very beginning of the Feature selection phase through HYSGA-GOA, all the necessary parameters of both algorithms are defined along with their specific values. All these parameters are recorded in tabular format and are shown in .

Table 1.

**Table 1:** HYSGA-GOA Configurational Parameters.

Simulation Parameters	Values
Workload	STO, NET, DEPL
Feature Selection	A hybrid of YSGA and GOA
No of Population	50
No of Iteration	100
No of Clusters (YSGA)	4
beta (YSGA)	2
Cmin(GOA)	0.1
Cmax(GOA)	1

**Algorithm 1:**
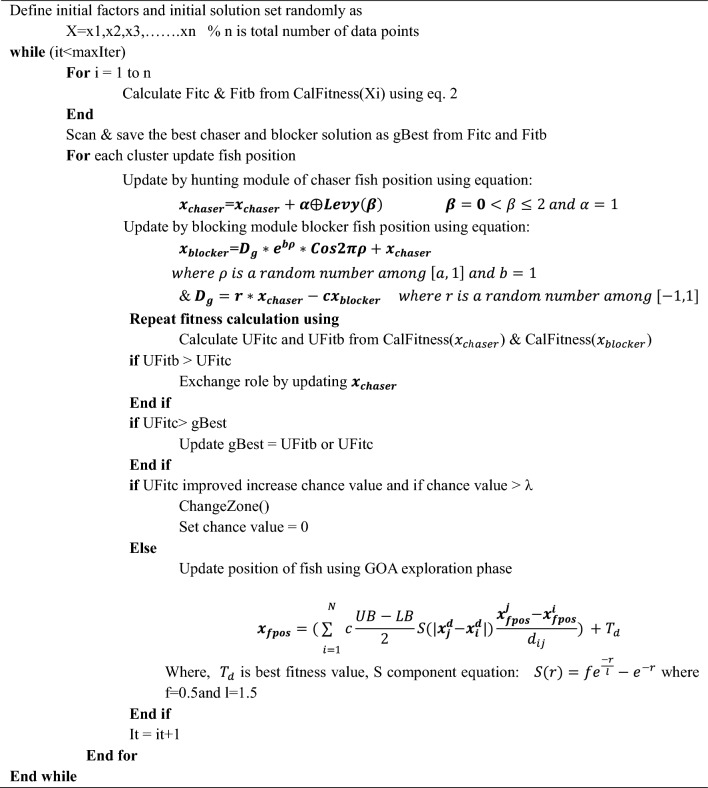
Feature Selection using HYSGA-GOA.

#### Initial YSGA phase

During this phase, a population of n goatfish is generated randomly that is uniformly distributed within the upper bound (UB) and Lower Bound (LB) values of dimensional search space. The initial position of the fish in the proposed work is determined by using the equation.

1 $${x}_{fpos}=rand\left(n,dim\right)*\left(UB-LB\right)+LB$$

Once the position of fishes is generated, fitness is calculated for each particle in terms of *purity* by using Eqs. 2 and 3.

2 $$Purity=\frac{1}{N}\sum \text{max}(classCount)$$

wherein,* N* represents the number of data points, *classCount* represents an array comprising count for each class label in a cluster, and *max(classCount)* refers to the maximum count in an array of *classcount.*

3 $$fitness= \left(1-Purity\right)$$

As mentioned earlier, YSGA hunts in groups. The clusters are formed in the algorithm by dividing the population. The elements of these clusters are formed by using the K-Means algorithm, wherein *K* represents the total number of clusters.

#### Chaser fish

Among the group of YSGA algorithm, there exists only one chaser fish which leads the hunting behaviour. The fish with the best fitness value is selected as the chaser fish in the algorithm as it is close to the solution. This chaser fish uses a random stroll to locate its target. The updated position of the chaser is evaluated by using Eq. 4.

4 $$x_{chaser} = x_{chaser} + \alpha \oplus Levy\left( \beta \right)$$

$$\beta =0<\beta \le 2 and \alpha =1$$

where ${x}_{chaser}$ and $\alpha$ determines the new position of chaser fish and step size whose value is 1 in our work. Moreover, “⨁” and “$\beta$” represent element-wise multiplication and Levy index respectively.

#### Blocker fish

Once the chaser fish is selected, the remaining fish in the YSGA Algorithm assume the role of *blocker fish*. The movement pattern of the blocker fish is represented by a logarithmic spiral, characterized by a gradual increase in distance from the origin. The blocker fish follows a spiral path around the chaser fish, with each blocker fish having its distinct path. These paths are updated and altered after each iteration, allowing the blocker fish to adapt their movement and maintain their role as obstacles to guide the exploration and search process of the algorithm. The updated position of the blocker fish is calculated by using Eq. 5.

5 $$x_{blocker } = D_{g} \times e^{b\rho } \times Cos2\pi \rho + x_{chaser}$$

wherein $\rho$ represents the random number among [a,1] and *b* = *1* determines the shape and direction of the spirals formed around chaser fish. With the increase in the number of iterations, the value of a start decreases from -1 to -2. ${D}_{g}$ represents the distance between the present location of chaser fish and blocker fish in a particular cluster and can be calculated by using Eq. 6.

6 $${D}_{g}=r*{x}_{chaser}-{cx}_{blocker}$$

wherein, “*r*” depicts the random number within the value of -1 to 1.

#### Roles exchange

Upon selecting the chaser and blocker fish, the chaser fish is identified as the one in closer proximity to the prey. The primary aim of the blocker fish is to prevent the prey from escaping. As the prey moves within the hunting area, if it comes close to a blocker fish, that particular blocker fish assumes the role of the chaser fish, while the previous chaser fish becomes a blocker fish. This role exchange phenomenon is known as a role swap. Within the algorithm, the chaser fish is determined based on the element with the highest fitness, ensuring its capability to effectively pursue and capture the prey.

#### Zone changing

The fish group proceeds to another area to look for fresh prey after fully utilizing the region by hunting every prey. The “*Changeof zone”* function is utilized in the proposed work to perform the change zone whenever necessary. In each zone-changing block, the fitness value for the new position is calculated. If the fitness value is better than the global best solution, then values are exchanged. By doing so, the tendency of the algorithm to get trapped in local minima is avoided.

#### GOA exploration

If there is no best solution found in the zone-changing phase, then *exploration factor (c*) is calculated for the GOA algorithm, based on its current location. This step is performed on every cluster for a particular number of iterations. The position of fish is updated by GOA by iterating over each fish position in each cluster. The updated position of the fish using GOA is given by Eq. 7.

7 $${x}_{fpos}=\left(\sum_{i=1}^{N}c\frac{UB-LB}{2}S(\left|{x}_{j}^{d}{-x}_{i}^{d}\right|)\frac{{x}_{fpos}^{j}{-x}_{fpos}^{i}}{{d}_{ij}}\right)+{T}_{d}$$

wherein ${T}_{d}$ and *S* represents the fitness value and component equation, given by: ${S(r)=fe}^{\frac{-r}{l}}-{e}^{-r}.$ The value of the current iteration and fitness value is displayed and the final best fitness value for the feature is stored. This process is repeated for a given number of iterations and finally, only discriminative and crucial features are selected from the given dataset.

### Classification using NN

**Figure 2:**
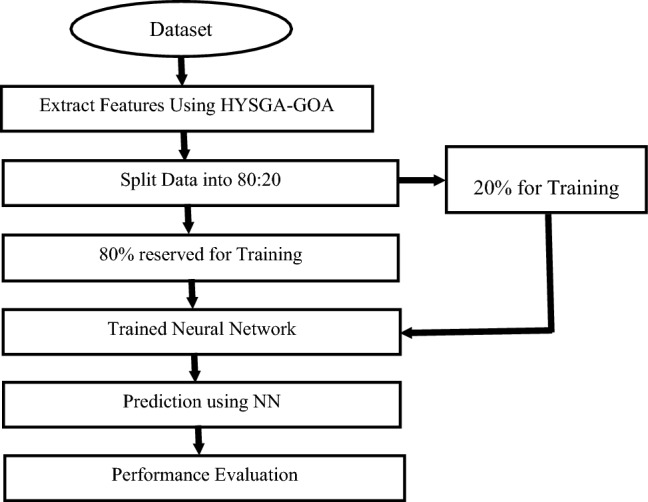
Flowchart of HYSGA-GOANet.

After selecting the features, it is time to predict software failures in cloud computing and for this, neural networks (NN) have been used in the proposed work. The flowchart of the proposed HYSGA-GOANet is shown in Fig. 2.

The selected features are divided into two categories of training and testing data in the ratio of 80:20. Here, 80% of the featured data is used for training the model while the remaining 20% is used for testing its efficacy. The featured data is then parsed to Neural Network for classification purposes which predicts the software failures in a cloud environment. In this section, we aim to describe the NN-based technique for predicting faults. Neural Networks are a type of computational model inspired by the structure and function of biological neural networks in the human brain. They are composed of interconnected nodes, called neurons, organized in layers. They are capable of learning complex nonlinear relationships and can handle high-dimensional data effectively. They can generalize from training examples to make accurate predictions on unseen data. By utilizing the power of NN, it becomes possible to analyze intricate patterns and relationships in the data to make accurate predictions regarding the occurrence of failures. The process begins by preparing a dataset that encompasses relevant features and labels indicating the presence or absence of failures. This dataset is then used to train the Neural Network, allowing it to learn from the patterns and dependencies present in the data. During training, the Neural Network automatically extracts meaningful features and optimizes its weights and biases to minimize prediction errors.

Once the Neural Network is trained, it is deployed for prediction purposes. New data related to the cloud software system is fed into the NN, which processes the information through its layers to generate predictions on the likelihood of failures. These predictions enable proactive actions, such as timely maintenance, resource allocation, and system management, aiming to prevent or mitigate software failures in the dynamic and complex cloud computing environment. By leveraging the capabilities of Neural Networks, the prediction of software failures in the cloud becomes more accurate and enables efficient decision-making for ensuring the reliability and stability of cloud-based applications. The efficacy of the proposed model on testing data is examined and validated through a range of experiments, whose detailed description is given in the next section of this research.

## Experimental study

This section of the paper presents the outcomes achieved with the proposed software failure detection model. The model utilizes a hybrid Feature Selection Technique (YSGA and GOA) along with a Neural Network (NN) classification technique to predict software failures in a cloud environment, employing MATLAB Software. The dataset used in this study is sourced from GitHub and is publicly available.

### Dataset Information

We have employed Failure-Dataset-OpenStack in the proposed work that is easily available on GitHub. This dataset incorporates the occurrences documented in the OpenStack cloud computing platform throughout three separate fault-injection campaigns conducted with three distinct workloads of DEPL, NET, and STO. The database represents three entirely different fault-injection campaigns in three separate folders, whose information is given below.

- **SEQ.tsv:** This folder represents a matrix with N rows and d columns that collected events during fault-injection experiments. Here, N represents the fault injection and d denotes the different types of event types respectively.
- **LCS_with_VMM.tsv:** it is a matrix made of N rows and 2d columns that comprises anomalies recognized by the LCS along with the VMM technique during fault injection experiments. *“N”* and *“d”* depict the fault injections and various event types. Moreover, the spurious anomalies are depicted by the first d columns i.e., 1 to “*d”* in the repository, and missing anomalies were depicted by the last *“d”* columns i.e., *d* + *1* to *2d* respectively.
- **Failure_Labels.txt:** A document holding the label class linked to each trial using fault injection. A number is used to represent the class label.

The workload applied throughout the fault-injection operations determines the number “*N”* of trials and the total number *“d”* of events. Therefore, it must be kept in mind that the matrix dimension in each folder's tsv files changes. Furthermore, the three workloads i.e., DEPL, NET, and STO experienced 6, 4, and 4 failures respectively.

### Performance metrics

In the proposed work, we have calculated the performance of the proposed approach with three different workloads, by employing a hybrid Feature selection technique and NN for classification. Experimental results were evaluated in terms of purity value that signifies the degree to which the predicted failure instances belong to a single class. It measures the quality and accuracy of the model's predictions by evaluating the consistency of the assigned labels. A higher purity value indicates a stronger correlation between the predicted failures and the actual failures in the specified class, demonstrating the model's ability to accurately classify failure instances. The purity value is calculated by using the Eq. 2. The purity value ranges from 0 to 1, where 1 represents a perfect clustering or classification result with all instances assigned to their correct classes, and 0 indicates the opposite, where all instances are assigned to different classes without any coherence.

### Performance analysis

**Figure 3:**
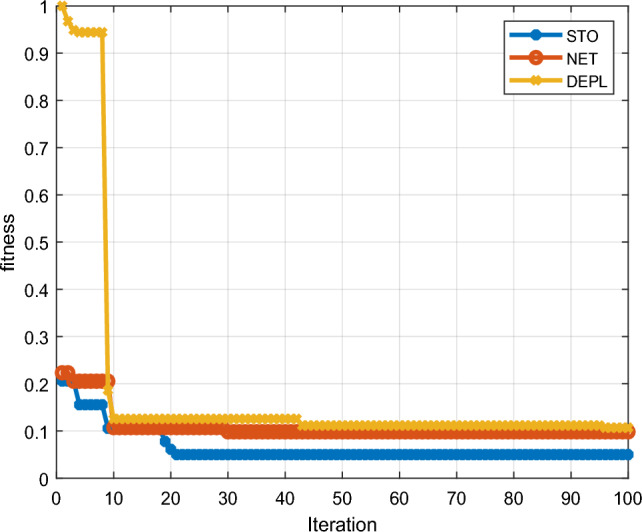
Fitness value for different workloads.

The proposed model's effectiveness is first evaluated and validated by analysing its fitness curve across three different workloads. In Fig. 3, a comparative graph illustrates the iterations on the x-axis and the corresponding fitness values on the y-axis. Upon careful examination of the graph, it is evident that the fitness value for the DEPL workload starts initially high as the model undergoes training. However, as the iterations progress, the fitness curves gradually decrease, indicating effective training of the model. Similar trends are observed for the NET and STO workloads, where the fitness values start slightly high and progressively decrease with increasing epochs. Among the three workloads, the STO workload demonstrates the lowest fitness value, followed by the NET and DEPL workloads, respectively.

### Comparative analysis for STO workload

**Figure 4:**
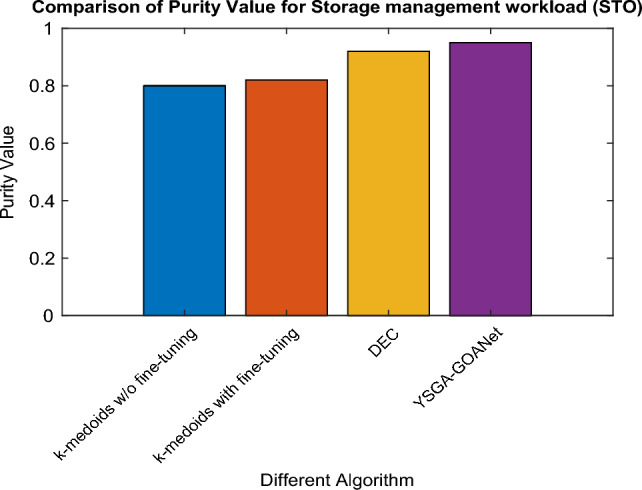
Comparison of purity in STO workload.

The efficacy of the proposed YSGA-GOANet approach is examined and validated by comparing it with conventional approaches in terms of their purity value for STO workload. The graphical results obtained for the same is shown in Fig. 4. Upon analysing the graph, it becomes evident that the K-medoids approach without fine-tuning achieves the lowest purity value of only 0.8. However, when fine-tuning is applied to the K-medoids model, the purity value slightly improves to 0.82. Further improvement is observed with the standard DEC model, which achieves a purity value of 0.92. Nevertheless, there remains room for enhancement. Remarkably, the proposed approach achieves a purity value of 0.95, representing a significant increase of 0.15, 0.13, and 0.03 compared to the K-medoids without fine-tuning, K-medoids with fine-tuning, and DEC models, respectively. The specific values of purity are recorded in tabular format and are shown in Table 2.

**Table 2:** Specific purity values for STO.

S no	Algorithm	STO Purity Value
1	K-medoids without fine-tuning	0.8
2	K-medoids with fine-tuning	0.82
3	DEC	0.92
4	Proposed YSGA-GOANet	0.95

### Comparative analysis for NET workload

**Figure 5:**
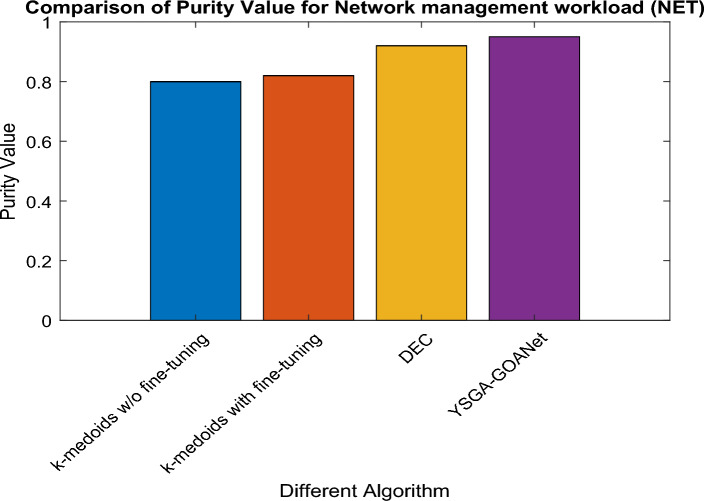
Comparison of purity in NET workload.

Similarly, the proposed model's functionality is tested and compared to standard approaches specifically for the NET workload, focusing on their purity values as illustrated in Fig. 5. Upon careful analysis of the graph, it is observed that the K-medoid approach without fine-tuning achieves a purity value of 0.8, which is consistent with the results for the STO workload. However, there is a slight improvement when applying the standard K-medoid model with fine-tuning, resulting in a purity value of 0.85. Furthermore, the DEC model demonstrates even better performance with a purity value of 0.96. Interestingly, in the case of the NET workload, the purity value experiences a decrease to 0.90179, indicating lower performance compared to the STO workload. The specific values obtained for purity in NET workload are recorded in Table 3.

**Table 3:** Specific purity values for NET.

S no	Algorithm	NET Purity value
1	K-medoids without fine-tuning	0.8
2	K-medoids with fine-tuning	0.85
3	DEC	0.86
4	Proposed YSGA-GOANet	0.90179

### Comparative analysis for DEPL workload

**Figure 6:**
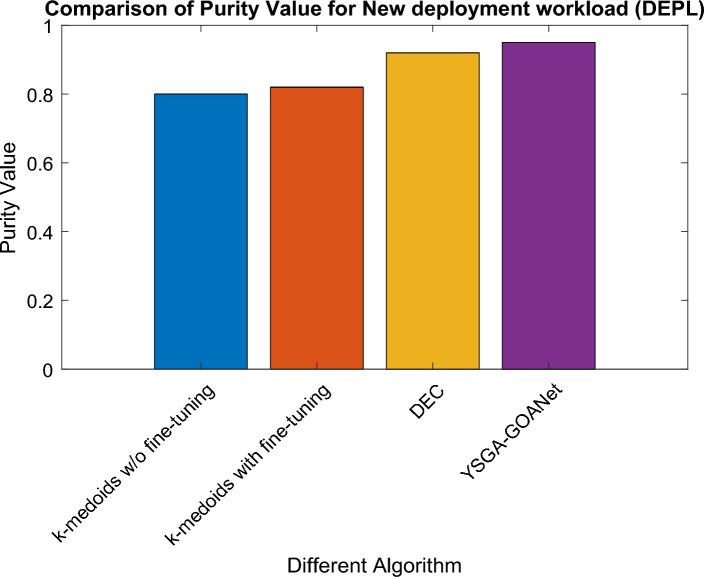
Comparison of purity for DEPL Workload.

To demonstrate the efficacy of our approach, we conducted a performance comparison with standard models in terms of their purity value specifically for the DEPL workload. The comparative graph is depicted in Fig. 6. Upon careful analysis of the graph, it becomes evident that the overall purity value is decreased for the DEPL workload due to the presence of data variability and the complexity of features. Notably, the graph showcases that the purity value for the k-medoids approach without fine-tuning was 0.7, while the k-medoids model with fine-tuning achieved a slightly improved purity value of 0.74. However, the DEC approach yielded a purity value of only 0.86 for the DEPL workload. Remarkably, our proposed approach outperforms all other comparable models, achieving an effective purity value of 0.89 for the DEPL workload. The specific value of purity achieved for the DEPL workload is given in Table 4.

**Table 4:** Specific purity values for DEPL.

S no	Algorithm	DEPL purity value
1	K-medoids without fine-tuning	0.7
2	K-medoids with fine-tuning	0.74
3	DEC	0.86
4	Proposed YSGA-GOANet	0.89302

### Findings from results

The analysis of purity values across all three workloads, namely STO, NET, and DEPL, reveals interesting findings. In the STO workload, the proposed approach demonstrates significant improvement over the k-medoids without fine-tuning, k-medoids with fine-tuning, and the standard DEC model, achieving a purity value of 0.95, with an increase of 0.15, 0.13, and 0.03 respectively. Similarly, in the NET workload, the proposed approach outperforms k medoids w/o and with fine-tuning along with DEC approaches, achieving a purity value of 0.90179 and an improvement of 0.101, 0.051, and 0.4 respectively. However, in the DEPL workload, while the proposed approach maintains its effectiveness with a purity value of 0.89123, it exhibits a slight decrease compared to the STO and NET workload. The purity value was increased by around 0.7, 0.15, and 0.03 for k-medoid without and with fine-tuning and DEC model. Overall, the findings demonstrate that the proposed approach consistently delivers superior results in terms of purity values, showcasing its effectiveness in accurately classifying software failures across different workloads.

## Conclusion

In the software industry, ensuring high quality is paramount, and early identification and prediction of software vulnerabilities play a vital role in achieving this objective. This research utilizes a hybrid approach that combines the Yellow Saddle Goat Fish Algorithm and the Grasshopper Optimization Algorithm for feature selection, along with the utilization of Neural Networks for classification. The efficacy of the proposed system was validated on the Failure-dataset-OpenStack repository using MATLAB software in terms of Purity values on three different workloads. The proposed model was able to yield a purity value of 0.95 on the STO workload whereas, this value was 0.901 and 0.89 on the NET and DEPL workloads respectively. These results signify that the proposed approach has the highest purity value for the STO workload, followed up by the NET and then the DEPL workload respectively. The proposed system provides valuable insights for cloud system designers and administrators to proactively identify and mitigate potential failures, thereby ensuring the continuity and reliability of cloud-based applications. However, the limitation of current system is that neural network is used that may lead to overfitting issues if data is complex or when training data is limited. Future research can focus on exploring additional optimization algorithms, incorporating other Deep learning techniques, and considering the dynamic nature of cloud environments to further enhance the accuracy and effectiveness of such prediction systems.

## Data Availability

The datasets used for experimentation and analysis in the current study can be accessed via the following web links: https://github.com/dessertlab/Failure-Dataset-OpenStack
